# Locally advanced breast cancer treated with neoadjuvant chemotherapy: Is breast-conserving surgery feasible?

**DOI:** 10.1016/j.amsu.2020.12.039

**Published:** 2020-12-26

**Authors:** Awad Ali Alawad, Rashid Ibrahim, Hossam Nawara, Adel Kheder, Sabry Abounozha

**Affiliations:** a University of Medical Sciences and Technology, Khartoum, Sudan; b University Hospitals Plymouth NHS Trust, Plymouth, UK; c University Hospital Southampton NHS Trust, Southampton, UK; d Northumbria Healthcare NHS Foundation Trust, Northumbria, UK

**Keywords:** Breast cancer, Neoadjuvant chemotherapy, Breast-conserving surgery

## Abstract

A best evidence topic has been constructed using a described protocol. The three-part question addressed was: is breast-conserving surgery feasible after neoadjuvant chemotherapy for locally advanced breast cancer?

Using the reported search, 19 articles were found, out of these 6 studies were deemed to be suitable to answer the question. The outcomes assessed were local recurrence rate. The best evidence showed that breast conserving surgery is safe in terms of local recurrence.

## Highlights

• Locally advanced breast cancer.
• Neoadjuvant chemotherapy.
• Breast-Conserving surgery.
• Local recurrence.

## 1 Introduction

This BET was designed using a framework outlined by the International Journal of Surgery [1]. This format was used because a preliminary literature search suggested that the available evidence is of insufficient quality to perform a meaningful meta-analysis. A BET provides evidence-based answers to common clinical questions, using a systematic approach of reviewing the literature.

## 2 Clinical scenario

A breast surgical resident is about to consent a 55-year-old female with locally advanced breast cancer (LABC) treated with neoadjuvant chemotherapy (NCT) for breast-conserving surgery (BCS). The patient is genuinely concern about the risk of local recurrence, and she is wondering if the procedure is associated with low recurrence rate?

## 3 Three-part question

Does [breast-conserving surgery following neoadjuvant chemotherapy] affects [the recurrence rate] in patients with [LABC]?

## 4 Search strategy

A. Embase 2002 to October 2020 using the OVID interface:

[Locally advanced breast cancer] AND [neoadjuvant chemotherapy] AND [breast-conserving surgery OR mastectomy] AND [recurrence].

B. Medline using the PubMed interface:

[Locally advanced breast cancer] AND [neoadjuvant chemotherapy] AND [breast-conserving surgery OR mastectomy] AND [recurrence].

The results were limited to English articles and human studies.

## 5 Search outcome

We identified 231 potentially relevant articles. After exclusion of duplicate references, nonrelevant literature, 19 candidate articles were considered. After careful review of the full text of these articles, 6 studies were identified to provide the best evidence to answer the question.

## 6 Result: see the table

**Table:** 

Author, date of publication, journal and country	Study type	Patient group	Outcomes Follow up	Key results	Additional comments
Cho JH, 2013 [2] Journal of Surgical oncology Korea	Retrospective Cohort Study, level III	Total of 1994 patients were randomized in two groups: **Group 1**: initial BCS **Group 2**: BCS after NCT **Group 3**: mastectomy after NCT	Primary endpoint: Local recurrence (LR) 5-year follow up	(LR) rate was: **Group 2** = 10.4% **Group 3** = 9.2%	-Single centre, -Large sample size -Long period of follow up
Eugene, 2006 [3] Int J Radiat Oncol Biol Phys USA	Retrospective Cohort Study, level III	Total of 815 patients underwent NCT and surgery 1. BCT group 2. Mastectomy group	Primary endpoint: Local recurrence (LR) Median follow-up was 120 months	The 10-year LR rates were extremely low and similar between the mastectomy and BCT groups	-Single centre -Large sample size over all -Non-randomized -Retrospective study
Parmar V, 2006 [4] Int J Surg India	Retrospective Cohort Study, level III	A total of 664 patients underwent NCT followed by surgery **Group1**: BCT **Group2**: Mastectomy	Local recurrence Mean follow-up of 36 months	Local recurrence rate was: **Group1**: 8% **Group2**: 10.7% (P < 0.001).	Short period of follow up
Rouzier R, 2006 [5] Cancer. France	Retrospective Cohort Study, level III	Total 594 patients. After NCT, 287 (48%) patients were eligible for BCT and 307 patients underwent a mastectomy.	LR rates were similar in patients treated with BCT and mastectomy.	Local recurrence rate was: BCT: 9% Mastectomy:8.7% Mean follow-up of 48 months	-Single centre -Large sample size -Retrospective
Shin HC, 2013 [6] Ann Surg Oncol Korea	Retrospective Cohort Study, level III	166 patients underwent BCS or mastectomy after NCT (NCT group) and 193 patients underwent surgery first (surgery group) in clinical stage III breast cancer patients.	The 5-year LR-free survival rates were 93.6% in the NCT group and 95.9% in the surgery group (P = 0.108).	In the NCT group, the 5-year LR-free survival rates were 96.3% in the mastectomy group, 94.7% in the preplanned BCS group and 90.9% in the downstaged BCS group (P = 0.669).	-Single centre, -Small sample size -Retrospective - Shorter follow-up -No randomization
Sweeting RS, 2011 [7] Am Surg USA	Retrospective cohort study, level III	122 patients underwent NCT. 44% patients had BCT and 56% mastectomy.	Local recurrence rate Median follow- up is **76.8** months	Disease-free survival was better for patients achieving BCT, with 5-year disease-free survival rates of 82% (95% CI, 69–90%) compared with 58% (95% CI, 45–69%) for mastectomy (P = 0.03).	-Single centre, -Small sample size, -Retrospective

## 7 Discussion

It is well known that neoadjuvant chemotherapy can effectively downsize the locally advanced breast tumors [8]. For patients with large tumors justifying mastectomy at the initial diagnosis, the use of NCT has been shown to downstage the primary tumor and make breast-conserving surgery feasible.

The two main goals of the surgeon when performing BCS are to obtain tumor-free margins and achieve a good cosmetic outcome by keeping the amount of healthy breast tissue excision as low as possible. Tumor-involved margins increase the risk of LRR and therefore require additional local therapy, such as a radiation therapy boost, re-excision, or even mastectomy.

In 2006, Rouzier et al. [5] developed a nomogram for breast cancer patients who receive NCT to predict residual tumor size and whether the patients could become eligible for BCS following neoadjuvant chemotherapy.

In our review, we investigated local recurrence rates after BCS compared with mastectomy in LABC patients having treated primarily with NCT. The main challenge for patients with LABC treated with BCS following NCT is to show satisfactory local recurrence rate compared to those treated with mastectomy. There are concerns that locally advanced tumors treated with BCS may have higher local recurrence rates than those treated with mastectomy after NCT because tumors treated with NCT may dwindle into local micrometastasis. This response is the main barrier to applying routine BCS in patients receiving NCT due to the difficulty of assessing surgical margins accurately [7]. The oncologic safety of BCS after NCT in patients with an initial diagnosis of LABC has been investigated in previous studies [2,3,9].

Breast-conserving surgery was found to be associated with a lower local recurrence than following mastectomy in some studies [2,4,10]. This probably does not really represent a true impact of extent of surgery, rather the inherent selection bias that discriminates between women who were responders (hence, offered breast conservation) and those who were non-responders (and therefore underwent mastectomy). Therefore, a careful clinical and radiological assessment after surgery is essential to ensure eligibility for BCT.

In our review, all studies showed no statically significant difference in the rate of local recurrence among the two types of surgery. However, these studies have some limitations such as short follow-up and lack of randomization.

## 8 Clinical bottom line

According to the above articles, the best evidence showed that BCT is feasible and oncologically safe after tumor downstaging by NCT in patients with locally advanced breast cancer.

## Limitation of this review

1. We are aware that the rather small sample size and the retrospective study design are limitations of our study. Despite these limitations, we believe our results to be clinically meaningful.
2. Single centre studies in most of the papers.
3. Shorter period of follow in some articles.

## Ethical approval

Not applicable.

## Source of funding

No funding

## Author contribution

AA: conducted the literature search and wrote the paper.

RI: assisted in the literature search and writing of paper.

HN: assisted in writing of paper.

AK: assisted in writing of paper.

SA: assisted in the literature search, editing of writing.

## Research registration number

1. Name of the registry:

2. Unique Identifying number or registration ID:

3. Hyperlink to your specific registration (must be publicly accessible and will be checked):

## Guarantor

Awad Alawad.

## Provenance and peer review

Not commissioned, externally peer-reviewed.

## Declaration of competing interest

None.
